# Circulating myocardial microRNAs from infarcted hearts are carried in exosomes and mobilise bone marrow progenitor cells

**DOI:** 10.1038/s41467-019-08895-7

**Published:** 2019-02-27

**Authors:** Min Cheng, Junjie Yang, Xiaoqi Zhao, Eric Zhang, Qiutang Zeng, Yang Yu, Liu Yang, Bangwei Wu, Guiwen Yi, Xiaobo Mao, Kai Huang, Nianguo Dong, Min Xie, Nita A. Limdi, Sumanth D. Prabhu, Jianyi Zhang, Gangjian Qin

**Affiliations:** 1 Department of Cardiology, Union Hospital, Tongji Medical College, Huazhong University of Science and Technology, Wuhan, 430022 China; 2 Department of Biomedical Engineering, University of Alabama at Birmingham, School of Medicine and School of Engineering, 35294 Birmingham, AL USA; 3 Department of Cardiology, Huashan Hospital, Fudan University, Shanghai, 200040 China; 4 Department of Cardiovascular Surgery, Union Hospital, Tongji Medical College, Huazhong University of Science and Technology, Wuhan, 430022 China; 5 Division of Cardiovascular Disease, Department of Medicine, University of Alabama at Birmingham, School of Medicine, Birmingham, 35294 AL USA; 6 Department of Neurology and Epidemiology, University of Alabama at Birmingham, School of Medicine, Birmingham, 35294 AL USA

## Abstract

Myocardial microRNAs (myo-miRs) are released into the circulation after acute myocardial infarction (AMI). How they impact remote organs is however largely unknown. Here we show that circulating myo-miRs are carried in exosomes and mediate functional crosstalk between the ischemic heart and the bone marrow (BM). In mice, we find that AMI is accompanied by an increase in circulating levels of myo-miRs, with miR-1, 208, and 499 predominantly in circulating exosomes and miR-133 in the non-exosomal component. Myo-miRs are imported selectively to peripheral organs and preferentially to the BM. Exosomes mediate the transfer of myo-miRs to BM mononuclear cells (MNCs), where myo-miRs downregulate CXCR4 expression. Injection of exosomes isolated from AMI mice into wild-type mice downregulates CXCR4 expression in BM-MNCs and increases the number of circulating progenitor cells. Thus, we propose that myo-miRs carried in circulating exosomes allow a systemic response to cardiac injury that may be leveraged for cardiac repair.

## Abstract (web summary)

During myocardial infarction, microRNAs (myo-miRs) are released into the circulation. Here Cheng et al. show that myo-miRs carried in exosomes are transferred to the bone marrow (BM), downregulate expression of CXCR4 in mononuclear cells, and increase the number of circulating BM progenitor cells, thus orchestrating a systemic response to myocardial injury.

## Introduction

Mobilization of progenitor cells (PCs) and other accessory cells from bone marrow (BM) to ischemically-injured heart is a physiological reparatory response^1^. Over the last 15 years, a large number of cell-therapy clinical trials have been conducted using BM PCs and demonstrated beneficial effects for ischemic heart disease^2^. However, the efficacy remains modest, and a better mechanistic understanding of BM PC trafficking and recruitment is needed for developing newer and more effective therapeutic strategies.

MicroRNAs (miRNAs) are bioactive small non-coding RNAs, which interact with the complementary sequences in the 3′ untranslated region (3′UTR) of protein-coding mRNAs, resulting in the inhibition of protein translation or mRNA degradation^3^. It is well known that some miRs are tissue-specifically expressed. For example, miR-208a and miR-499-5p are highly enriched in the heart tissue, while miR-1a and miR-133a are abundantly expressed in both heart and skeletal muscles^4–6^. These myocardial abundant miRs (hereto referred as myo-miRs) have been shown to be markedly elevated in the peripheral blood (PB) following acute myocardial infarction (AMI) in patients and animals^7^. However, how these myo-miRs are transported in the circulation and what their biological significance is remains largely unknown.

Exosomes are small lipid-bilayer vesicles, with a 50–150 nm diameter, that are released by healthy and diseased cells^8^. Accumulating evidence suggests that exosomes mediate exchanges of genetic materials, DNA fragments, mRNAs and miRs, between cells^8^. However, whether these actions of exosomes play a role in the systemic response to cardiac ischemic injury has not been explored.

Here we investigated the role of circulating myo-miRs and exosomes in mice with AMI. We found that following cardiac injury, myo-miRs are rapidly released in a remarkable quantity to the PB where they are carried primarily in the exosomes. The exosomal myo-miRs are transferred selectively to other tissues and preferentially to the BM mononuclear cells (MNCs), in which they suppress CXCR4 expression and mediate PC mobilization. Thus, our studies reveal a novel pathway of systemic response to cardiac ischemic injury, which may be leveraged for cell based cardiovascular repair.

## Results

### Myo-miRs are markedly elevated in PB after AMI and efficiently transferred into BM-MNCs

**Fig. 1:**
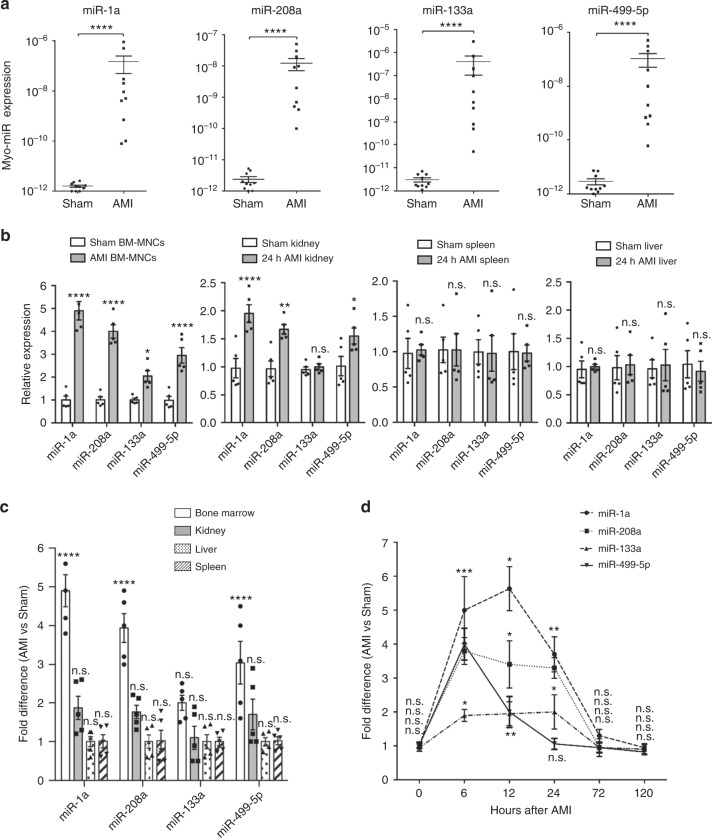
Myo-miRs are released into PB following AMI and transported into BM-MNCs. AMI and Sham surgeries were performed in C57BL/6 mice; then at various time points, the plasma, BM-MNCs, and different organs were isolated and subjected to qRT-PCR analyzes of myo-miRs, miR-1a, 208a, 133a, and 499-5p. **a** myo-miR levels in the plasma 6 h post-surgery (*n* = 10 animals per group). **b** myo-miR levels in the BM-MNCs, kidney, spleen, and liver 12 h post-surgeries, expressed relative to the levels in the intact controls (*n* = 5 animals per group), and **c** the fold difference in AMI vs. Sham mice (*n* = 5 animals per group). *p < 0.05, **p < 0.01, ***p < 0.001, ****p < 0.0001 vs. Sham; n.s., no significant. **d** The fold difference of myo-miR levels in the BM-MNCs of AMI vs. Sham mice at 0, 6, 12, 24, 72, and 120 h post-surgery. *p < 0.05 **p < 0.01, ***p < 0.001 vs. Sham at same time point. *n* = 5 animals per group per time point. An unpaired t test was used in **a** and a two-way ANOVA was used in **b**, **c**, and **d** for statistical analysis. Error bars represent mean ± s.e.m

We surgically induced AMI in mice and 6 h later, isolated plasma for measuring myo-miRs with quantitative RT-PCR (qRT-PCR). The levels of the four myo-miRs, miR-1a, miR-133a, miR-208a, and miR-499-5p, were markedly (~10^4^–10^5^ times) higher in AMI mice than in Sham-operated mice **(**Fig. 1a). We then analyzed myo-miR uptakes by different organs; while the levels of myo-miRs in the liver and spleen were similar between the two treatment groups, their levels in BM-MNCs and kidney were significantly higher in AMI mice than in Sham mice (Fig. 1b). The fold change was the greatest in BM-MNCs (Fig. 1c). It is unlikely that the increase of myo-miRs was due to their upregulation in the BM-MNCs themselves by ischemia, because their expression levels in these cells are extremely low and unaltered by hypoxia treatment (Supplementary Figure 1). These results suggest that myo-miRs released from the infarcted heart are transferred rather selectively to different organs and more efficiently into the BM cells. Furthermore, we analyzed the time-course of myo-miR accumulation in the BM-MNCs, which peaked between 6 and 12 h post-AMI, decreased at 24 h, and returned to basal level by 72 h (Fig. 1d).

### Exosomes mediate transfer of circulating myo-miRs into BM-MNCs

**Fig. 2:**
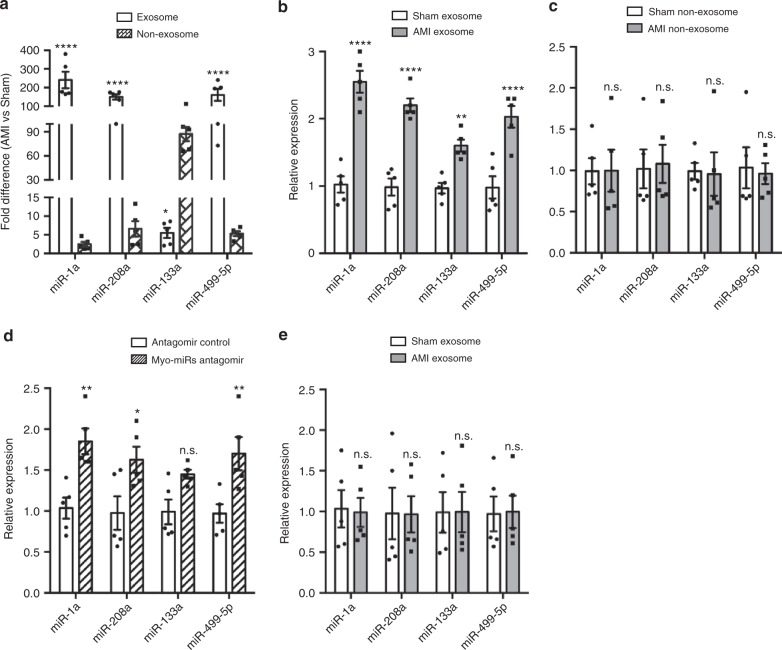
Exosomes mediate the transfer of circulating myo-miRs into BM-MNCs. Exosomes and the non-exosomal component were isolated from the plasma of AMI and Sham mice 6 h post-surgery. **a** The levels of myo-miRs in the exosomes and the non-exosomal component were analyzed by qRT-PCR and expressed as fold difference in AMI vs. Sham mice. *p < 0.05, ****p < 0.0001 vs. Sham. *n* = 5 animals per group. **b**, **c** In vitro, freshly-isolated mouse BM-MNCs (2 × 10^7^/well) were treated for 12 h with the exosomes (20 μg) (**b**) or non-exosomal component (150 μl) (**c**) from AMI or Sham mice; then, the levels of myo-miRs in the BM-MNCs were quantified with qRT-PCR. **p < 0.01, ****p < 0.0001 vs. treatment with Sham exosomes or Sham non-exosomal component; n.s. not significant. *n* = 5 biologically independent samples per group. **d**, **e** In vivo, exosomes (40 μg in 300 μL PBS/mouse) (**d**) or the non-exosomal component (300ul/mouse) (**e**) from AMI or Sham mice was *i.v*. injected into intact C57BL/6 mice; 12 h later, the levels of myo-miRs in the recipient BM-MNCs were quantified via qRT-PCR. *p < 0.05, **p < 0.01 vs. treatment with Sham exosomes or Sham non-exosomal component; n.s., not significant. *n* = 5 animals per group. A two-way ANOVA was used for statistical analysis. Error bars represent mean ± s.e.m

To investigate whether myo-miRs are carried in exosomes in the circulation, we isolated exosomes from the mouse plasma and collected exosome-depleted component. The exosomes were characterized by electron microscopy, NanoSight, and Western blotting (Supplementary Figure 2). Then we quantified the levels of myo-miRs in both exosomes and the exosome-depleted (non-exosomal) component. Compared to Sham mice, levels of the four myo-miRs in the circulating exosomes were significantly higher in AMI mice (Fig. 2a). Notably, these results were reproduced in humans, in which acute ST–elevation MI significantly increased circulating exosomal myo-miRs (Supplementary Figure 3). Further studies in mice revealed that miR-1, 208, 499 were elevated overwhelmingly in the exosomes, while miR-133 was increased predominantly in the non-exosomal component (Fig. 2a). These results suggest that miR-1, 208, and 499 are primarily, while miR133 is partially, carried by exosomes in the circulation.

Next, we investigated whether exosomes can mediate the transfer of myo-miRs to the BM. BM-MNCs were isolated from intact mice (Supplementary Figure 4) and treated with exosomal or non-exosomal component from AMI or Sham mice. qRT-PCR analyzes were performed with the six reference miRNAs stably expressed in the plasma as control since their expression in the BM-MNCs was not altered by hypoxia treatment (Supplementary Table 1 & Supplementary Figure 5). The levels of myo-miRs were significantly higher in BM-MNCs treated with exosomes from AMI mice than from Sham mice (Fig. 2b), while no difference was observed between BM-MNCs treated with non-exosomal components from AMI and Sham mice (Fig. 2c). Then, we i.v. injected intact mice with exosomes or the non-exosomal component isolated from AMI or Sham mice. Consistently, myo-miRs were marked increase in the BM-MNCs of mice treated with exosomes (Fig. 2d) but not mice treated with non-exosomal component (Fig. 2e) from AMI mice. These data suggest that it is the exosomal, not the non-exosomal, component of the plasma that mediates the transfer of circulating myo-miRs into BM-MNCs after AMI.

Further, we investigated whether the exosomes themselves could be transferred to the BM cells. Exosomes were isolated from the plasma of AMI or Sham mice (6 h post-surgery), labeled with a fluorescent membrane marker PKH67, and i.v. injected into intact mice; 12 h later, BM-MNCs were isolated for analysis of their uptake of exosomes by flow cytometry. Injections of equal amount of AMI and Sham exosomes led to 92% and 89% PKH67-positive BM-MNCs in the recipient mice, respectively (Supplementary Figure 6). These results indicate that circulating exosomes are transferred efficiently to the BM cells and may regulate the function of BM PCs.

### Myo-miRs downregulate CXCR4 expression in BM-MNCs in vitro

**Fig. 3:**
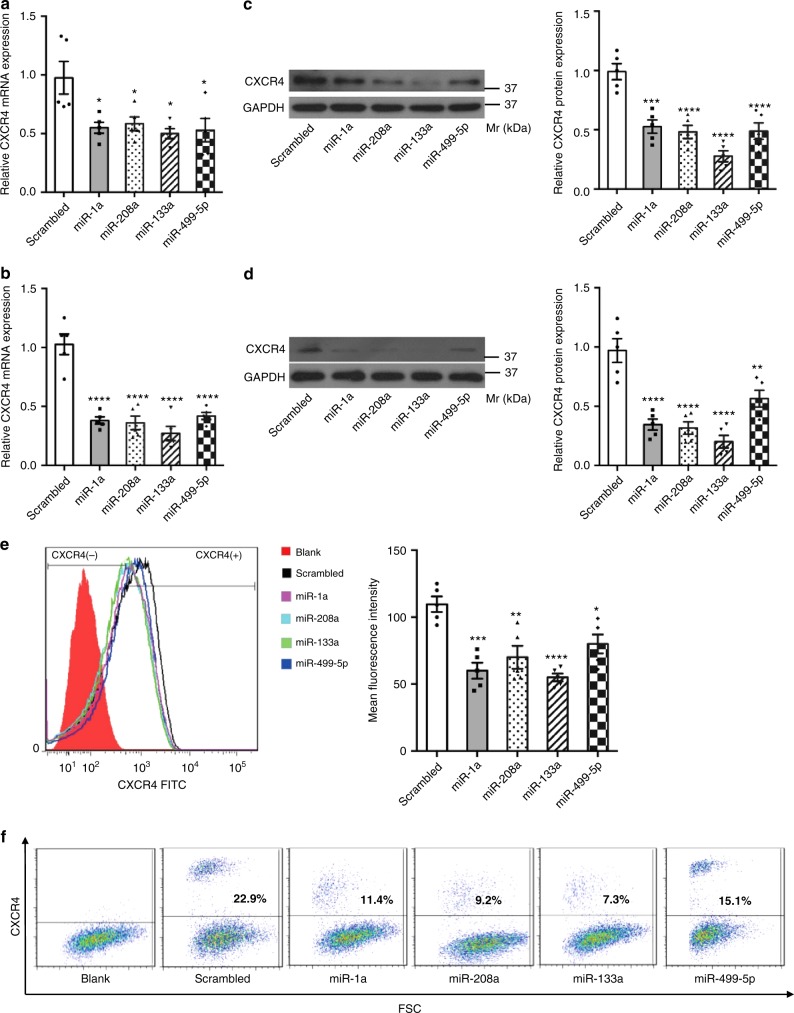
Myo-miRs downregulate CXCR4 expression in BM-MNCs and MSCs in vitro. BM-MNCs (2 × 10^6^/well) and MSCs (0.5 × 10^6^/well) were transfected with synthesized miR-1a, miR-208a, miR-133a, or miR-499-5p mimics or with a scrambled miR (final concentrations: 50 nM); 24 h later, CXCR4 mRNA expression in the BM-MNCs (**a**, **c**, **e**) and MSC (**b**, **d**, **f**) was quantified by RT-PCR (**a**, **b**), Western-blotting (**c**, **d**), and flow cytometry (**e**, **f**). *p < 0.05, **p < 0.01, ***p < 0.001, ****p < 0.0001 vs. Scrambled. *n* = 5 biologically independent samples per group. A one-way ANOVA was used for statistical analysis. Error bars represent mean ± s.e.m

Ample evidence from us and other laboratories suggests that PCs are retained in the BM by interactions between the CXC chemokine stromal cell-derived factor 1 (SDF-1) and CXC chemokine receptor 4 (CXCR4)^9^. Blockade of CXCR4 in the BM mediates mobilization of BM PCs to circulation and contribute to ischemic repair^10^. Since myo-miRs carried by exosomes were transferred into BM-MNCs and our bioinformatic analyzes identified a putative binding site of miR-1a at position 295-302 (-ACAUUCCA-) of the 3′ UTR of CXCR4 (Supplementary Figure 7a), we investigated whether these miRs have a role in the regulation of CXCR4 expression. We first transfected individual myo-miR mimics in both freshly isolated BM-MNCs and BM culture-derived mesenchymal stem cells (MSCs). Surprisingly, overexpression of any single myo-miR was able to downregulate CXCR4 expression in both BM-MNCs and MSCs, as evaluated by qRT-PCR, Western blotting, and flow cytometry analyzes (Fig. 3). In contrast, overexpression of two skeletal muscle specific miRs, miR-208b and miR-133b, did not alter the levels of CXCR4 (Supplementary Figure 8). Furthermore, dual-luciferase activity reporter assays confirmed that miR-1a targets the 3’UTR at position 295-302 (Supplementary Figure 7b). However, miR-133a, miR-208a or miR-499-5p did not regulate the CXCR4 3′ UTR reporter activity (Supplementary Figure 7c), suggesting that they may regulate CXCR4 expression through mechanisms unrelated to the 3′UTR. Collectively, these data suggest that myo-miRs inhibit CXCR4 expression in BM-MNCs and MSCs in vitro, implicating a potential function in BM PC mobilization.

### Exosomal transfer of myo-miRs downregulates CXCR4 in BM-MNCs

**Fig. 4:**
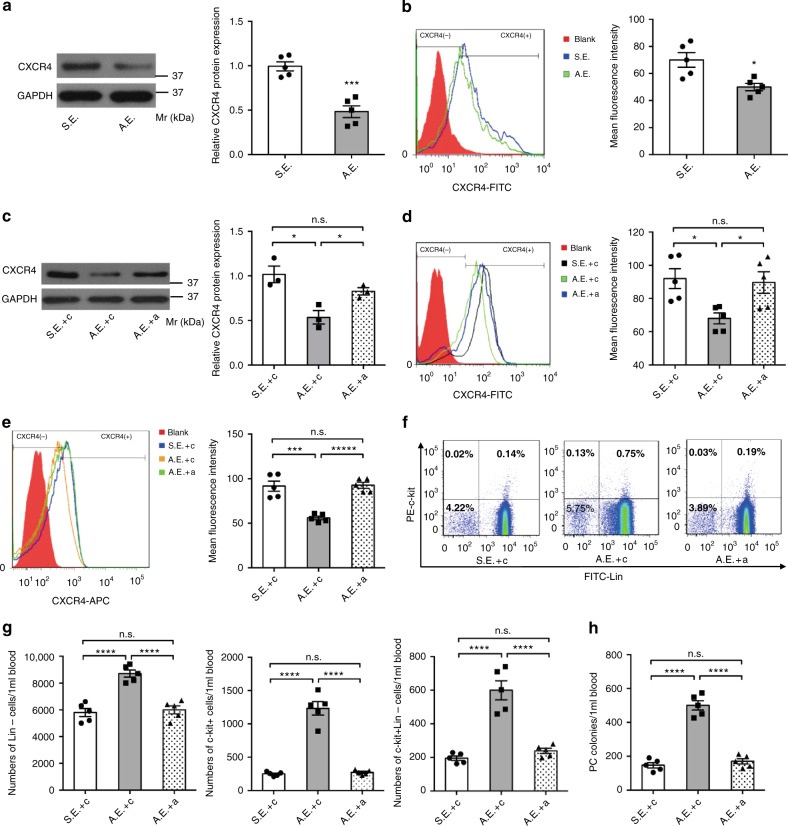
Exosomal transfer of myo-miRs downregulates CXCR4 in BM-MNCs and induces BM PC mobilization. Exosomes were isolated from the plasma of AMI mice (AMI-exosomes, A.E.) or Sham mice (Sham-exosomes, S.E.) 6 h post-surgery. **a**–**d** In vitro, BM-MNCs (2 × 10^7^/well) were cultured for 24 h with A.E. (20ug), S.E. (20 μg), A.E. plus 10 nM non-targeting scrambled control sequence (**c**) (A.E. + c), S.E. + c, or A.E. plus 10 nM (4 × 2.5 nM each) antagomir (A.E. + a), then quantified for CXCR4 expression by Western-blotting (**a**, **c**; *n* = 5 biologically independent samples per group for **a**, *n* = 3 biologically independent samples per group for **c**) and flow-cytometry (**b**, **d**, *n* = 5 biologically independent samples per group). *p < 0.05, ***p < 0.001. **e**–**h** In vivo, C57BL/6 mice were injected with a (80 mg [20 mg each] antagomir/kg body weight/day) or **c** (80 mg/kg/day) for 3 consecutive days, then subjected to AMI or Sham surgery and 6 h later, to isolation of plasma exosomes. The isolated exosomes were subsequently i.v. injected into intact C57BL/6 mice (40 μg diluted in 300 μL PBS/mouse) and 12 h later, BM-MNCS and PB-MNCs were isolated from the recipient mice for assessments of PC mobilization. **e**–**g** Flow cytometry analyzes for CXCR4 expression in BM-MNCs (**e**), percentages of c-kit^+^, Lin^–^, and c-kit^+^Lin^–^ cells in the PB-MNCs (**f**), and calculation of absolute c-kit^+^, Lin^–^, and c-kit^+^Lin^–^ cell numbers per 1 ml PB (**g**). **h** The colony-forming PCs in the PB-MNCs were evaluated via colony formation assay. ***p < 0.001, ****p < 0.0001, n.s., no significant. *n* = 5 animals per group. An unpaired t test was used in (**a**, **b**) and a one-way ANOVA was used in (**c**–**h**) for statistical analysis. Error bars represent mean ± s.e.m

Next, we investigated whether exosome-mediated myo-miR transfer regulates the expression of CXCR4. BM-MNCs were cultured with exosomes isolated from AMI or Sham mice for 24 h. The expression of CXCR4 was significantly reduced in BM-MNCs treated with exosomes from AMI mice but not with exosomes from Sham mice (Fig. 4a, b). Co-treatment with myo-miRs specific inhibitors abolished the AMI-exosomes mediated downregulation of CXCR4 expression (Fig. 4c, d), indicating that the effect was specific to myo-miRs.

### Exosomal transfer of myo-miRs induces mobilization of BM PCs

Finally, we investigated whether exosome-mediated downregulation of CXCR4 contributes to BM PC mobilization. Exosomes were isolated from AMI and Sham mice that had been pre-treated with anti-myo-miRs antagomirs or non-targeting scrambled oligonucleotides for 3 days (Supplementary Figure 9), and i.v. injected into intact mice; 12 h later, BM and PB MNCs in the recipients were analyzed. The level of CXCR4 expression was significantly reduced in the BM-MNCs of mice treated with exosomes from AMI mice (AMI-exosomes), while the reduction was diminished if the exosome donor mice were pretreated with antagomirs (Fig. 4e). Notably, c-kit^+^, Lin^–^, and Lin^–^c-kit^+^ cells in the PB were markedly increased in mice treated with AMI-exosomes but not with Sham-exosomes or AMI-exosomes from mice with antagomir pre-treatment (Antagomir-AMI-exosomes) (Fig. 4f, g). Importantly, we further performed PC colony formation assays and confirmed that the colony-forming PCs in the PB were also increased in mice treated with AMI-exosomes but not with Sham-exosomes or Antagomir-AMI-exosomes (Fig. 4h). Collectively, our results indicate that the exosome-mediated myo-miRs transfer downregulates CXCR4 expression in BM-MNCs and contributes to BM PC mobilization.

## Discussion

In this study, we have identified a previously unknown mechanism by which the ischemically-injured myocardium “signals” to mobilize BM PCs. We found that following MI, circulating exosomes and their cargo myo-miRs were transferred selectively to peripheral organs and preferentially to the BM in an exosome-dependent manner. Notably, the transferred myo-miRs downregulate CXCR4 expression in the BM cells, resulting in PC mobilization.

Although it is well-recognized that exosomes externally-prepared from stem cells have beneficial effects of on cardiovascular repair^11,12^, whether endogenous exosomes represents a physiologically relevant regulatory mechanism in the cardiovascular system remains undefined. Our work reveals that exosomes released from the ischemic heart are in fact mediating the systemic response of BM PCs to the site of injury. These observations are compelling, as circulating myo-miRs are not only a bio-marker, but also a functional constituent of the intricate crosstalk between ischemic heart and the remote BM organ. Because mobilization of PCs and other accessory cells from BM to peripheral circulation is a crucial step in the systemic response to cardiac ischemic injury and plays an important role in the turnover of vascular endothelium and restoration of coronary function^8^, exploitation of this mechanism may have the potential to enhance ischemic cardiovascular repair.

It is known that exosomes mediate exchange of genetic materials between cells; the bilayer lipid structure of exosomes protects miRs from degradation and maintains their integrity. A previous report showed that exosomes mediate transfer of miR-1 into kidney and urine^13^. In this study we found exosomes mediate transfer of myo-miRs to BM. Intriguingly, miR-1, 208, and 499 are primarily, while miR133 is partially, carried by exosomes in the circulation. Others have reported that in addition to being packed into exosomes or microvesicles, extracellular miRNAs can also be transferred by high-density lipoprotein (HDL)^14,15^ or bound by Argonaute2 (AGO2) protein complex^16^. Whether miR133a is transported in other forms in circulation is still unknown at present and warrants further investigation in the future. In addition, we found that the level of miR133a in the circulation is highest among the four myo-miRs post AMI, and that it suppresses CXCR4 expression to a greater extent than the other three myo-miRs. Thus, despite miR133a being only partially carried by exosomes, it may function potently.

The underlying mechanisms for the sorting of specific miRs into exosomes remain largely unclear^17^. Recent studies reveal that sumoylated hnRNPA2B1 recognizes the GGAG motif in the 3′ portion of miRNA sequences and causes specific miRNAs to be packed into exosomes; however no binding motif has been identified^18^. Others discovered that the 3’ ends of uridylated endogenous miRNAs are mainly presented in exosomes derived from B cells or urine, whereas the 3′ ends of adenylated endogenous miRNAs are mainly presented inside B cells^19^. The changes in miRNA-repressible targets levels that occur in response to cell activation may cause miRNA sorting to exosomes, partially by differentially engaging them at the sites of miRNA activity and exosome biogenesis^20^. Thus, specific sequences present in certain miRNAs may guide their incorporation into exosomes^21^.

The SDF-1/CXCR4 signal pathway is central to PC mobilization. We and others have previously shown that CXCR4 antagonist AMD3100 can induce BM PC mobilization and improve cardiovascular repair after MI^9,10^, and that the downstream effect of SDF-1/CXCR4 axis is mediated by Src family kinase activity^9,22^. However, the endogenous regulators of CXCR4 has been largely unclear. In this study, we found that myo-miRs suppress CXCR4 expression in BM-MNCs and mediate BM PC mobilization to the circulation. Our results suggest that miRs released from ischemic heart are natural suppressants of CXCR4, thus representing a feedback circuit. A previous report suggested an miR-1a putative target site on CXCR4^23^; here we have verified that miR-1a directly targets on CXCR4 3′ UTR. Interestingly, despite that miR-133a, miR-208a, and miR-499-5p suppress CXCR4 mRNA and protein expression, our bioinformatics analyzes revealed no binding site of these 3 miRNAs on the gene, and neither of them regulates the 3′ UTR reporter activity; thus it is likely that these 3 myo-miRs regulate CXCR4 expression through mechanisms unrelated to the 3′ UTR.

Existing evidence suggest that myo-miRs may also regulate PC differentiation^24^. For example, miR-1 has been shown to enhance the angiogenic differentiation of human cardiomyocyte progenitors^25^, and over-expression of miR-1 improves the effectiveness of MSC transplantation in the infarcted heat^26^. MiR-499 has been shown to induce cardiac differentiation of rat MSCs through Wnt/β-catenin signaling pathway^27^. Most interestingly, combined injection of miR-1, 133, 208, and 499 to the ischemic myocardium is capable of inducing the reprogramming and conversion of fibroblasts to cardiomyocyte-like cells and improves heart function^28,29^. These observations suggest that, in addition to promoting BM PC mobilization, exosomal myo-miRs may also modify the potency of PCs^30^.

A weakness of this study is that we did not examine those exosomes from non-myocardial origin, such as endothelial and platelet miRNAs. It is possible that some miRNAs from other cell-types in the injured heart may also contribute to the BM response, which remains to be tested in the future. Similarly, due to the scope of this current study, we did not evaluate the functional significance of this systemic myo-miRs-SDF1/CXCR4 pathway on PC-mediated cardiac repair, which might be challenging to differentiate myo-miRs’ BM PC effects from their direct cardiac effects^31^.

In conclusion, our data demonstrate that circulating exosomal myo-miRs released from the ischemic heart can mediate BM PC mobilization by targeting CXCR4 expression. Because of the critical role of BM PCs in the ischemic tissue repair, the mechanism identified in this study could be an important component of the physiological response to ischemic injury and thus could be leveraged for treatment of ischemic heart disease.

## Methods

### Mouse care and AMI surgery

All animal experiments in this report were approved by the Animal Care and Use Committee of Huazhong University of Science and Technology and performed in compliance with the “Guide for the Care and Use of Laboratory Animals” (NIH publication) and all relevant ethical regulations for animal testing and research. Male, 8 week-old C57BL/6 mice were used for all the experiments unless specified. For surgical induction of AMI, the mice were anesthetized by intraperitoneal injection of sterile pentobarbital sodium (50 mg/kg body weight, Sigma-Aldrich, St. Louis, MO, USA). After endotracheal intubation and mechanical ventilation, the chest of mouse was open by a left intercostal thoracotomy. AMI was induced by permanent ligation of the left anterior descending coronary artery (LAD) as we previously described^32^. The Sham-operated animals went through all the procedures except LAD ligation. Post-operative care was performed by following our approved animal study protocol. For pain management, Metacam was subcutaneously injected (1 mg/kg) at the end of surgery and continued twice daily for 3 days.

### Human studies

Human studies were approved by the Institutional Review Board for Human Use (IRB) of the University of Alabama at Birmingham (protocols IRB-151201004 and IRB X130807012) and performed in adherence to the Belmont Report and Declaration of Helsinki. Informed consent was obtained from all subjects. Plasma samples were drawn from three patients (age range 49–74 yo; two males, one female) with underlying coronary artery disease and acute ST–elevation myocardial infarction during their initial hospital presentation, immediately prior to coronary intervention and stent placement. Control plasma samples were obtained from three patients (age range 64–78 yo; one male, two females) with underlying coronary artery disease (but without evidence of acute coronary syndrome) after elective percutaneous coronary intervention.

### Isolation and labeling of circulating exosomes

Exosomes were isolated from mouse and human plasma by following recommended protocols^33–35^. The plasma was centrifuged at 3000 × g for 15 min to remove cell debris and platelets. Then each 250 μL platelet-free plasma was added into 63 μL ExoQuick Exosome Precipitation Solution (System Biosciences Co., Ltd., USA) and carefully mixed. After 4 °C refrigeration for 30 min, the mixture was centrifuged at 1500 g for 30 min at 4 °C to sediment exosomes (20 μg exosomes/250 μL platelet-free plasma). The supernatant was collected as non-exosomal component. For comparison, exosomes and the non-exosomal component from equal amount of plasma were used. To facilitate tracking in vitro and in vivo, exosomes were labeled with PKH67 Green Fluorescent Cell Linker Kit (Sigma-Aldrich Co., Ltd., USA) by following the manufacturer’s protocol. The amounts of exosomes chosen for use in the in vitro and in vivo experiments were based on our previously report^36^.

### qRT-PCR analysis of myo-MiRs in the plasma and exosomes

Plasma RNAs were isolated from 250 μL plasma samples using 750 μL TRI reagent (TB126-200, Molecular Research Center, Inc.) according the manufacturer’s protocol. To extract RNAs from exosomes, the exosome pellet was suspended in 1 mL of TRIzol reagent (Invitrogen) and quantified by NanoDropTM 2000/2000c Spectrophotometers (ThermoFisher Scientific). Equal quantity (5 ng) of total RNAs from each sample was used for cDNA synthesis by using a PrimeScript® RT reagent kit (Takara). The reverse transcriptions of miRNAs were performed by looped miRNA-specific RT primers for myo-miRs (miR-1a, miR-208a, miR-133a and miR-499-5p) and reference miRNAs (miR-19b-3p, 103-3p, 154-5p, 200b-3p, 342-3p, and 434-3p) (Guangzhou RiboBio). The reference miRNAs are stably-expressed in the circulation as determined by miRNA sequencing of plasma collected from AMI and Sham mice (Supplementary Table 1). PCR reactions were performed in SYBR® Green Master Mix (Takara). The cycling program for miRNAs included a 20 s initial pre-incubation at 95 °C followed by 37 cycles of 95 °C for 10 s, 60 °C for 20 s, and 70 °C for 1 s. Real-time PCR was conducted in an ABI PRISM 7900 Sequence Detector system (AB Applied Biosystems). The Ct value was defined as the cycle number at which the fluorescence exceeded the threshold of 100 RFU. The plasma samples with similar Ct values (difference < 0.3) for each of all six reference miRNAs were considered to have same amounts of starting RNAs, and the Ct values of circulating myo-miRs in AMI and Sham samples with same amounts of starting RNAs were compared directly. All PCR primer sequences are reported in Supplementary Table 2.

### BM-MNC and PB-MNC isolation

About 1 ml PB was collected from each group by aspiration from heart, and BM was obtained by flushing the cavity of femurs and tibias. MNCs were obtained by density centrifugation using 1.083 g/ml Lymphocyte separation medium (MP Co., Ltd.), as we previously described^22,37^.

### Flow cytometry

Cells were blocked with 50% rat serum and mouse Fc blocker (BD Bioscience) for 10 min, then stained for 30 min with fluorophore-conjugated antibodies, anti-Lin-FITC (eBioscience; 17A2/RA3-6B2/M1-70/TER-119/RB6-8C5, 1:25), anti-c-kit-PE (eBioscience; 104D2, 1:25), anti-CD105-PE (eBioscience, MJ7/18, 1:25), anti-CD11b-FITC (eBioscience; M1/70, 1:25), anti-CD34-PE (Invitrogen; MEC14.7, 1:25), anti-CD45-PE (eBioscience; 30-F11, 1:25), or their corresponding isotype control antibodies (eBioscience). For CXCR4 staining, primary monoclonal CXCR4 antibody (abcam; EPUMBR3, 1:25) and FITC- or APC-conjugated secondary antibody (goat-anti-rabbit IgG; BD Bioscience) were used. The cells were firstly gated for the intact cell population using forward scatter versus side scatter plots. All flow cytometry data were acquired on an LSRII (BD Biosciences, CA) and analyzed with FlowJo (Treestar, OR).

### BM-MSC culture

BM-MSCs were obtained from mice as described previously^22^. Briefly, BM-MNCs were cultured in low glucose DMEM medium (HYCLONE Co., Ltd., USA) supplemented with 10% FBS (Gibco Co., Ltd., USA); 24 h later, the unattached cells were removed, and the attached cells were cultured continuously until 80%~90% confluence (referred as passage 0, P0). The P0 cells were then detached with trypsin-EDTA (HYCLONE Co., Ltd., USA) and split 1:2 or 1:3 into new plates as P1 cells. For the experiments reported in this study, BM-MSCs at P3-P5 were used after verification with flow cytometry analyzes for expression of MSC markers (i.e., CD44 and CD105) and absence of hematopoietic markers (i.e., CD34 and CD45).

### Western blotting

Proteins were extracted using RIPA Lysis Buffer (Thermo Fisher Scientific, MA, USA) containing protease inhibitors cocktail (Cell Signaling Technology, MA, USA). Protein concentration was measured using the BCA Protein Assay Kit (Beyotime Institute of Biotechnology). Then, protein extract (20 μg) was separated by SDS-PAGE and transferred onto polyvinylidene fluoride microporous membranes (Merck Millipore, Darmstadt, Germany). The membrane was blocked with 5% milk in 0.5% Tris-buffer saline solution (pH 7.6) for 1 h, then incubated overnight at 4 °C with primary antibodies for CXCR4 (abcam; UMB2, 1:1000), GAPDH (abcam; EPR16891, 1:5000), CD9 (abcam; EPR2949, 1:2000), CD63 (Santa Cruz; MX-49.129.5, 1:200), TSG101 (abcam; EPR7130(B), 1:1000), or actin (abcam; EPR16769, 1:5000). The membrane was then incubated at room temperature for 30 min with HRP-conjugated secondary antibodies. The image data were collected on Bio-Rad molecular Imager with Image Lab^TM^ Software and analyzed with NIH ImageJ.

### Overexpression of myo-miRs in BM-MNCs and BM-MSCs

Myo-miRs mimics were synthesized by RiboBio Co., Ltd. (Guangzhou, China) based on mouse mature miR sequences: miR-1a (miRBase IDs: MIMAT0000123), miR-208a (miRBase IDs: MIMAT0000520), miR-133a (miRBase IDs: MIMAT0000145) and miR-499-5p (miRBase IDs: MIMAT0003482). The transfection was carried out by using the riboFect™ CP Transfection Kit (RiboBio Co., Ltd.) according to the manufacturer’s instructions.

### Inhibition of myo-miRs

AntagomiRs are chemically modified anti-sense single-stranded RNA molecules complementary to the mature miRNAs, which can inhibit target miRNAs via degradation and are stable in vivo for at least 2 weeks^38^. MicrOFF™ myo-miRs (miR-1a, miR-208a, miR-133a and miR-499-5p) antagomirs and micrOFF™ miRNA antagomir Negative Control were synthesized by RiboBio Co., Ltd. The sequences of these myo-miRs antagomirs are: miR-1a antagomir, 5′-AUA CAU ACU UCU UUA CAU UCC A-3′; miR-208a antagomir, 5′-ACA AGC UUU UUG CUC GUC UUA U-3′; miR-133a antagomir, 5′-CAG CUG GUU GAA GGG GAC CAA A-3′; miR-499-5p antagomir, 5′-AAA CAU CAC UGC AAG UCU UAA-3′. The four antagomiRs were injected separately, with 1 h intervals, via the tail vein at a dose of 80 mg total antagomiRs (20 mg each in 75 μL volume)/kg body weight/day for 3 consecutive days according to the manufacturer’s instructions^39^. The antagomir negative control was administered at the same dose and injection intervals. The functional inhibition of myo-miRs by the administered antagomirs in vivo was verified by qRT-PCR of the myo-miRs’ known targets in the heart.

### PC colony-forming assay

The PC colony-forming assay was performed in 35-mm dishes with a semisolid methylcellulose medium containing SCF and other recombinant cytokines (MethoCult GF M3434, StemCell Technologies, Canada) by following the manufacturer’s protocol; 2 × 10^5^ PB-MNCs were seeded in each dish, and colonies were counted 12 days later, as we described previously^9^.

### Statistical analysis

All values are reported as mean ± s.e.m. Two-tailed Student’s *t*-test was used to compare two means. One-way or two-way analysis of variance (ANOVA) with a Bonferroni correction was used to compare multiple (>2) means with one or two independent variables, respectively. A *p*-value of <0.05 was considered significant.

### Reporting summary

Further information on experimental design is available in the Nature Research Reporting Summary linked to this article.

## Supplementary information

Supplementary Information

Reporting Summary

Source Data

## Data Availability

The microRNA-seq data is accessible at Gene Expression Omnibus (accession number: GSE124545). All remaining data are included in the article and Supplementary Information files, or available from the authors upon reasonable request.
