# Supplementary material for: Circulating myocardial microRNAs from infarcted hearts are carried in exosomes and mobilise bone marrow progenitor cells
Source: Nat Commun. 2019 Feb 27;10:959. doi: 10.1038/s41467-019-08895-7 (PMC6393447; doi:10.1038/s41467-019-08895-7)
Supplement: Supplementary file 1 — Supplementary Information [file 41467_2019_8895_MOESM1_ESM.pdf]

Supplementary Information

Circulating myocardial microRNAs from infarcted hearts are carried in exosomes and mobilise bone marrow progenitor cells

Cheng et al.

Supplementary Tables

Supplementary Table 1: Stably expressed miRNAs in the plasma

miRNA Symbol	Sham Group		MI Group	
	Sham1_S1-UMIs	Sham2_S2-UMIs	MI_1_S3-UMIs	MI_2_S4-UMIs
mmu-miR-103-3p	24509	15445	22289	28192
mmu-miR-1198-5p	6800	4243	6550	6884
mmu-miR-154-5p	192	132	184	213
mmu-miR-16-1-3p	210	174	195	238
mmu-miR-19b-3p	11021	7870	12149	14047
mmu-miR-200b-3p	9531	7442	8638	10587
mmu-miR-210-3p	1375	995	1553	1602
mmu-miR-30b-5p	10384	7531	12536	13708
mmu-miR-342-3p	18676	15361	18382	21504
mmu-miR-434-3p	2029	1660	2148	2245
Arithmetic Mean	8473	6085	8462	9922
Avg. of Arithmetic Mean	7279		9192	

For identification of miRNAs that are stably expressed in the plasma and unaltered by AMI, the plasma samples were collected from AMI and Sham mice 6 h post-surgery; then total RNA was isolated and sequenced by Next Gen miRNA Sequencing (n=2 biologically independent samples per group). MicroRNAs with best stability measures (Fold difference less than 1.5) are listed, and those marked in blue were used as reference miRNAs to normalize the analysis of myo-miRs in this report.

Supplementary Table 2: Sequences of PCR primers, miRNA mimics and antagomires

	Species	Name	miRBase IDs	Forward primers	Reverse primers
miRNA primers	mouse & human	mmu-miR-103-3p	MIMAT0000546	AGCAGCAUUGUACAGGGCUAUGA	
	mouse & human	mmu-miR-1198-5p	MIMAT0005859	UAUGUGUUCCUGGCUGGCUUGG	
	mouse & human	mmu-miR-154-5p	MIMAT0000164	UAGGUUAUCCGUGUUGCCUUCG	
	mouse	mmu-miR-16-1-3p	MIMAT0004625	CCAGUAUUGACUGUGCUGCUGA	
	mouse & human	mmu-miR-19b-3p	MIMAT0000513	UGUGCAAAUCCAUGCAAAACUGA	
	mouse & human	mmu-miR-200b-3p	MIMAT0000233	UAAUACUGCCUGGUAAUGAUGA	
	mouse & human	mmu-miR-210-3p	MIMAT0000658	CUGUGCGUGUGACAGCGGCUGA	
	mouse & human	mmu-miR-30b-5p	MIMAT0000130	UGUAAACAUCCUACACUCAGCU	
	mouse & human	mmu-miR-342-3p	MIMAT0000590	UCUCACACAGAAAUCGCACCCGU	
	mouse & human	mmu-miR-434-3p	MIMAT0001422	UUUGAACCAUCACUCGACUCCU	
	mouse & human	mmu-miR-1a-3p	MIMAT0000123	UGGAAUGUAAAGAAGUAUGUAU	
	mouse & human	mmu-miR-208a-3p	MIMAT0000520	AUAAGACGAGCAAAAAGCUUGU	
	mouse & human	mmu-miR-133a-3p	MIMAT0000145	UUUGGUCCCCUUCAACCAGCUG	
	mouse & human	mmu-miR-499-5p	MIMAT0003482	UUAAGACUUGCAGUGAUGUUU	
	human	hsa-miR-16-1-3p	MIMAT0004489	CCAGUAUUAACUGUGCUGCUGA	
mRNA primers	mouse	CXCR4	N/A	TCAGCCTGGACCGGTACCT	GCAGTTTCCTTGGCCTTTGA
	mouse	Myocd	N/A	TTCTGGGTTGTTAGCTGCTGTCCT	ATGTGCATAGTAACCAGGCTGGCA
	mouse	Kcnmb1	N/A	CTGGGAGTGGCAATGGTAGTG	CCGAGTGTCTTCCGTGTGATAC
	mouse	GAPDH	N/A	GCACAGTCAAGGCCGAGAAT	GCCTTCTCCATGGTGAA

For qPCR of miRNAs, stem-loop primers were used. The forward primer was designed based on the sequence of a specific miRNA. The reverse primer was designed based on the stem loop part of the RT primers, which is common for all miRNAs; they are proprietary products of the company, thus not provided.

Supplementary Figures

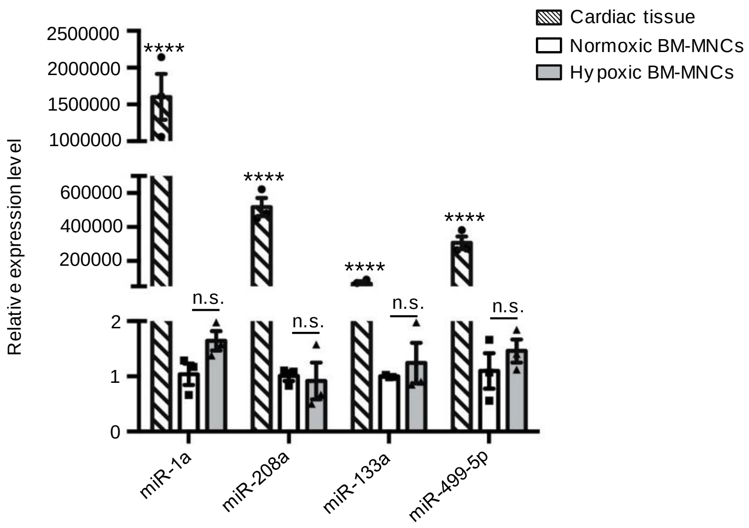

Supplementary Figure 1: Myo-miRs expression in normoxic and hypoxic BM-MNCs. Freshly-isolated mouse BM-MNCs ($2X10^7$/well) were cultured under normoxic or hypoxic (1% O_2) condition for 12 h; then, levels of myo-miRs were quantified with qRT-PCR and expressed relative to the values in Normoxic BM-MNCs. n=3 biologically independent samples. ****p<0.0001 vs. normorxic or hypoxic BM-MNCs. n.s., not significant. A two-way ANOVA was used for statistical analysis. Error bars represent mean ± s.e.m.

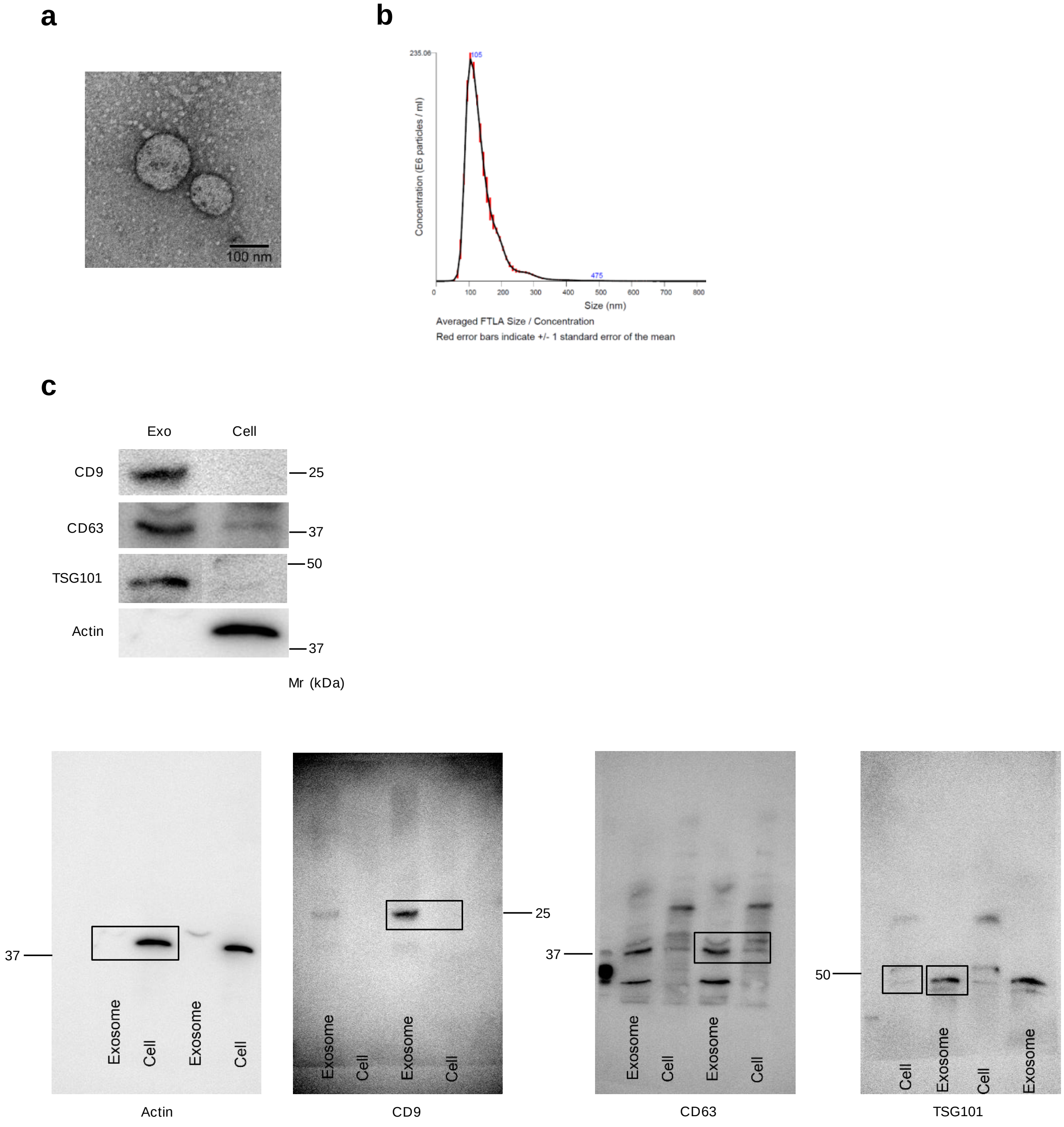

Supplementary Figure 2: Characterization of circulating exosomes isolated from mouse plasma. (**a**) Transmission electron microscope (TEM) image. (**b**) Nanoparticle tracking analysis of size distribution. (**c**) Western blotting analyses of exosome markers (*upper panel*, representative images; *lower panels*, original full blots, black frames indicating where the images were cropped). n=2 biologically independent samples. Cell, BM-MNCs; Exo, plasma exosomes.

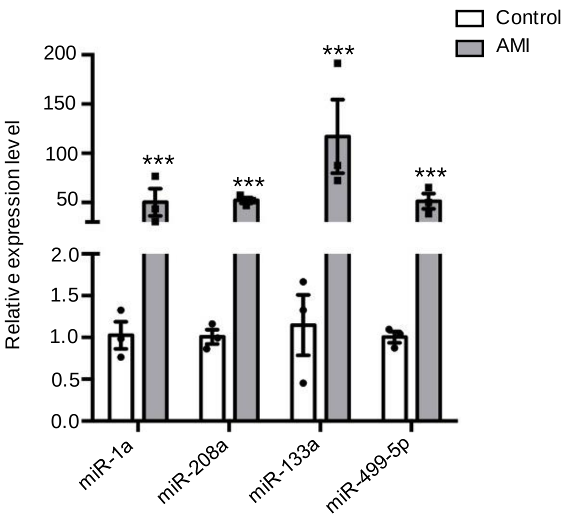

Supplementary Figure 3. The myo-miR levels in circulating exosomes are significantly higher in patients with acute ST–elevation MI than in patients without evidence of acute coronary syndrome. AMI plasma samples were drawn from three patients (age range 49 - 74 yo; 2 males, one female) with underlying coronary artery disease and acute ST–elevation MI during their initial hospital presentation, immediately prior to coronary intervention and stent placement. Control plasma samples were obtained from three patients (age range 64-78 yo; 1 male, 2 females) with underlying coronary artery disease (but without evidence of acute coronary syndrome) after elective percutaneous coronary intervention. About 600-800 µL plasma were used for isolation of exosomes, and myo-miRs in the exosomes were quantified as described in Methods and expressed relative to the values in control subjects. n=3 patients per group. ***$p<0.001$. A two-way ANOVA was used for statistical analysis. Error bars represent mean ± s.e.m.

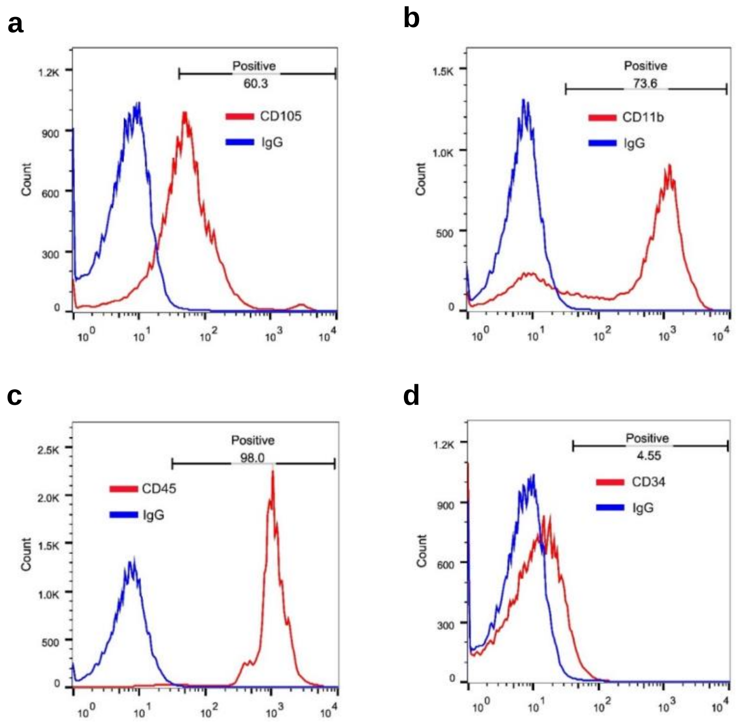

Supplementary Figure 4. Flow cytometry analyses of BM-MNCs. Freshly-isolated mouse BM-MNCs were analyzed by flow cytometry for the expression of surface markers, CD105 (**a**), CD11b (**b**), CD45 (**c**), and CD34 (**d**).

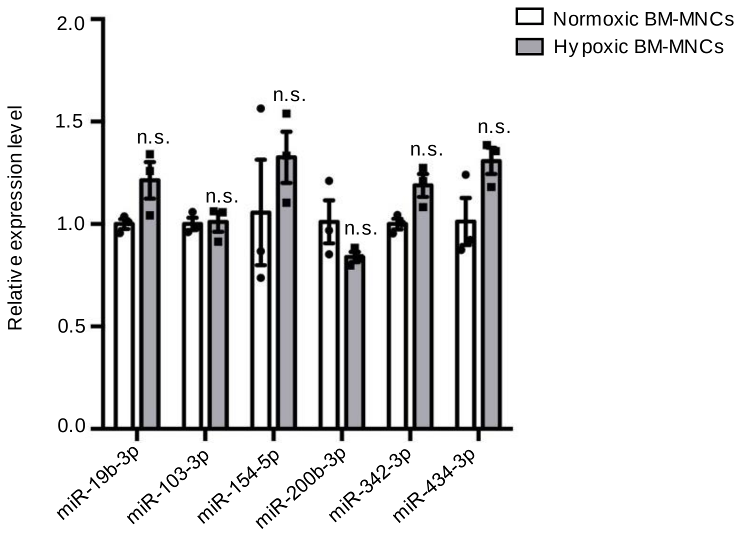

Supplementary Figure 5: The circulating reference miRNAs are also stably expressed in the BM-MNCs. Mouse BM-MNCs were isolated and cultured under normoxia or hypoxia (1% O_2) for 12 h. Then RNAs were isolated; and miRNAs expression were analyzed by qPCR, normalized to U6 RNA, and expressed relative to the values in normoxic BM-MNCs. n=3 biologically independent samples. n.s., not significant. A two-way ANOVA was used for statistical analysis. Error bars represent mean ± s.e.m.

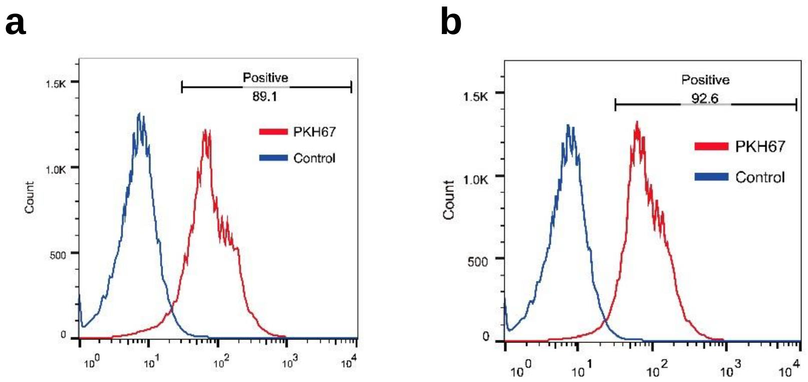

Supplementary Figure 6: Flow cytometry analyses of exosome internalization in BM-MNCs of recipient mice. The exosomes were isolated from plasma 6 h post Sham or AMI surgery, labeled with PKH67, and i.v. injected into intact C57BL/6 mice (40μg in 300μL PBS/mouse); 12 h later, BM-MNCs were isolated and analyzed by flow cytometry. Shown are representative of 3 independent experiments. (**a**) BM-MNCs from recipients of Sham exosomes. (**b**) BM-MNCs from recipients of AMI exosomes.

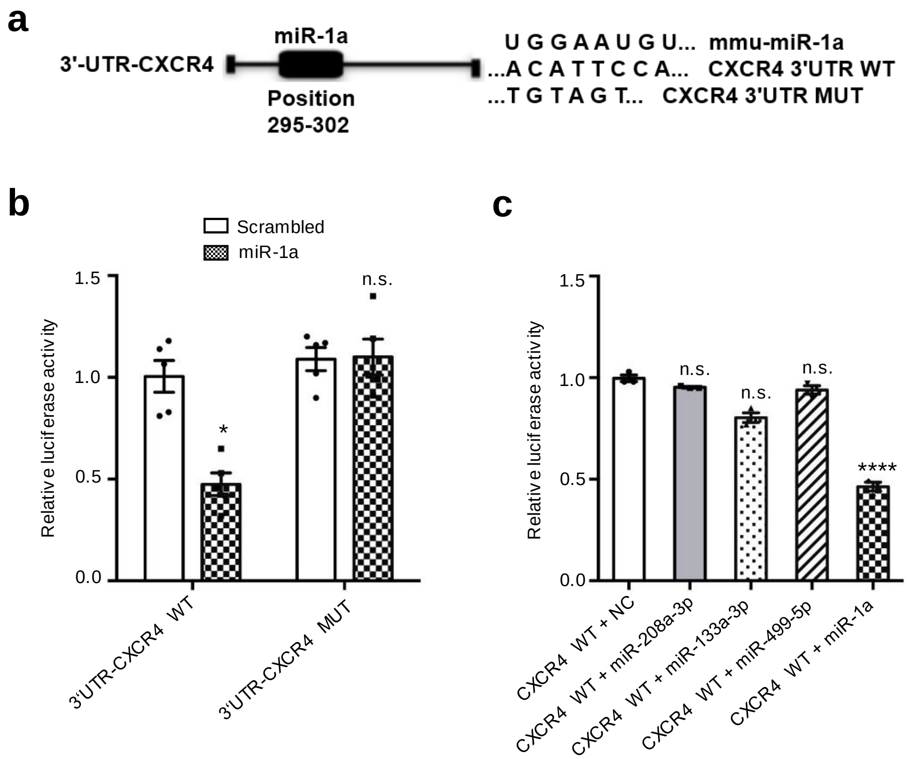

Supplementary Figure 7: The effects of myo-miRs on CXCR4 3'-UTR reporter activity. (**a**) Schematic representation of miR-1a targeting site at the 3'-UTR of mouse CXCR4. (**b**) NIH/3T3 cells were transiently transfected with a luciferase reporter construct carrying WT or mutant miR-1a seeding sequence and with miR-1a or control non-targeting scrambled oligonucleotide; 24 h later, the relative activity of firefly luciferase was measured and normalized to that of renilla luciferase. *p<0.05, n=5 biologically independent samples per group. (**c**) NIH/3T3 cells were transiently transfected with the luciferase reporter construct carrying WT CXCR4 3'-UTR and with miR-208a-3p, miR-133a-3p, miR-499-5p, miR-1a (positive control), or a non-targeting scrambled oligonucleotide (negative control, NC); 24 h later, the relative activity of firefly luciferase was measured and normalized to that of renilla luciferase. The values are expressed relative to the values in negative controls. n=3 biologically independent samples. n.s., no significant vs. CXCR4 WT + NC. ****p<0.0001 vs. CXCR4 WT + NC. A two-way ANOVA was used in **a** and a one-way ANOVA was used in **b** for statistical analysis. Error bars represent mean ± s.e.m.

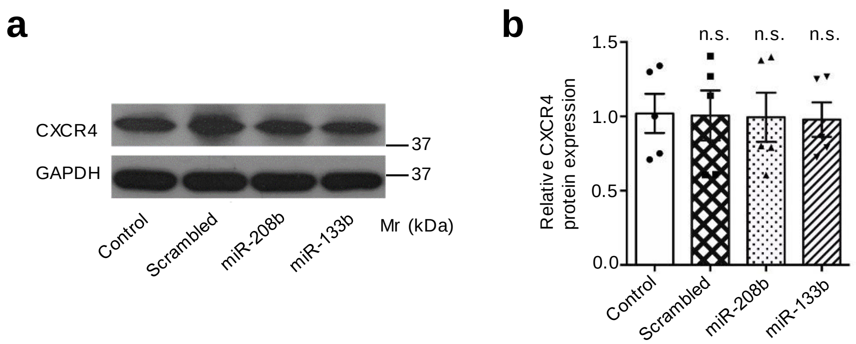

Supplementary Figure 8: Skeletal muscle specific miRNAs, miR-133b and miR-208, do not regulate CXCR4 expression in BM-MNCs. BM-MNCs were isolated from C57BL/6 mice and transfected with synthesized miR-133b or miR-208b mimics or a control scrambled oligonucleotide at a final concentration of 50 nM; 24 h later, CXCR4 expression was analyzed by Western blotting (**a**) and quantified densitometrically (**b**). n=5 biologically independent samples per group. n.s., no significant vs. control. A one-way ANOVA was used in **b** for statistical analysis. Error bars represent mean ± s.e.m.

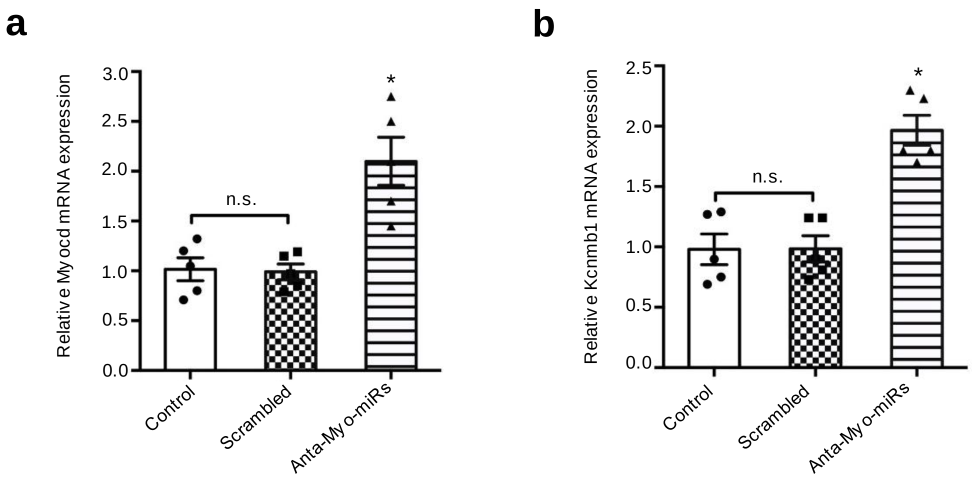

Supplementary Figure 9: C57BL/6 mice were injected with myo-miRs' antagomirs (80mg [20mg each]/kg body weight/day or control scrambled oligonucleotides (80mg/kg/day) for 3 consecutive days. Then the hearts were isolated and the levels of myocd (known mmu-miR-1a target) (**a**) and kcnmb1 (known mmu-miR-133a target) (**b**) were quantified via qRT-PCR. *$p<0.05$, $n=5$ animals per group. n.s., no significant. A one-way ANOVA was used for statistical analysis. Error bars represent mean ± s.e.m.
