# Supplementary material for: Circulating myocardial microRNAs from infarcted hearts are carried in exosomes and mobilise bone marrow progenitor cells
Source: Nat Commun. 2019 Feb 27;10:959. doi: 10.1038/s41467-019-08895-7 (PMC6393447; doi:10.1038/s41467-019-08895-7)
Supplement: Supplementary file 2 — Reporting Summary [file 41467_2019_8895_MOESM2_ESM.pdf]

nature research

Corresponding author(s): Gangjian Qin, Min Cheng

Last updated by author(s): Jan 12, 2019

Reporting Summary

Nature Research wishes to improve the reproducibility of the work that we publish. This form provides structure for consistency and transparency in reporting. For further information on Nature Research policies, see Authors & Referees and the Editorial Policy Checklist.

Statistics

For all statistical analyses, confirm that the following items are present in the figure legend, table legend, main text, or Methods section.

n/a	Confirmed	
☐	☒	The exact sample size (*n*) for each experimental group/condition, given as a discrete number and unit of measurement
☒	☐	A statement on whether measurements were taken from distinct samples or whether the same sample was measured repeatedly
☐	☒	The statistical test(s) used AND whether they are one- or two-sided<br>*Only common tests should be described solely by name; describe more complex techniques in the Methods section.*
☐	☒	A description of all covariates tested
☐	☒	A description of any assumptions or corrections, such as tests of normality and adjustment for multiple comparisons
☐	☒	A full description of the statistical parameters including central tendency (e.g. means) or other basic estimates (e.g. regression coefficient) AND variation (e.g. standard deviation) or associated estimates of uncertainty (e.g. confidence intervals)
☒	☐	For null hypothesis testing, the test statistic (e.g. F, t, r) with confidence intervals, effect sizes, degrees of freedom and P value noted<br>*Give P values as exact values whenever suitable.*
☒	☐	For Bayesian analysis, information on the choice of priors and Markov chain Monte Carlo settings
☒	☐	For hierarchical and complex designs, identification of the appropriate level for tests and full reporting of outcomes
☒	☐	Estimates of effect sizes (e.g. Cohen's d, Pearson's r), indicating how they were calculated

Our web collection on statistics for biologists contains articles on many of the points above.

Software and code

Policy information about availability of computer code

Data collection	Flow cytometry data were acquired on LSRII (BD Biosciences). Western blotting data were collected on Bio-Rad molecular Imager with Image LabTM Software. The PCR data were collected with ABI PRISM 7900 Sequence Detector system (AB Applied Biosystems).
Data analysis	Prism6 Software was used for statistical analysis. Flow cytometry data were analyzed with Flowjo (Treestar). Western blotting data were analyzed with NIH Imaging J.

For manuscripts utilizing custom algorithms or software that are central to the research but not yet described in published literature, software must be made available to editors/reviewers. We strongly encourage code deposition in a community repository (e.g. GitHub). See the Nature Research guidelines for submitting code & software for further information.

Data

Policy information about availability of data

All manuscripts must include a data availability statement. This statement should provide the following information, where applicable:

- Accession codes, unique identifiers, or web links for publicly available datasets
- A list of figures that have associated raw data
- A description of any restrictions on data availability

The microRNA-seq data is accessible at Gene Expression Omnibus (accession number: GSE124545 [https://www.ncbi.nlm.nih.gov/geo/query/acc.cgi?acc=GSE124545]). All remaining data are included in the article and Supplementary Information files, or available from the authors upon reasonable request.

Field-specific reporting

Please select the one below that is the best fit for your research. If you are not sure, read the appropriate sections before making your selection.

☒ Life sciences ☐ Behavioural & social sciences ☐ Ecological, evolutionary & environmental sciences

For a reference copy of the document with all sections, see nature.com/documents/nr-reporting-summary-flat.pdf

Life sciences study design

All studies must disclose on these points even when the disclosure is negative.

Sample size	Sample sizes were chosen based on previous experience with the animal models or following convention of the methods.
Data exclusions	No completed data were excluded from the experiments presented in this manuscript.
Replication	Data described in this manuscript were reliably reproduced.
Randomization	The animals were randomized to receive AMI or Sham surgery, antagomirs or scrambled treatment.
Blinding	The investigators were blinded to group allocation during data collection and analysis.

Reporting for specific materials, systems and methods

We require information from authors about some types of materials, experimental systems and methods used in many studies. Here, indicate whether each material, system or method listed is relevant to your study. If you are not sure if a list item applies to your research, read the appropriate section before selecting a response.

Materials & experimental systems

n/a	Involved in the study	
☐	☒	Antibodies
☒	☐	Eukaryotic cell lines
☒	☐	Palaeontology
☐	☒	Animals and other organisms
☐	☒	Human research participants
☒	☐	Clinical data

Methods

n/a	Involved in the study	
☒	☐	ChIP-seq
☐	☒	Flow cytometry
☒	☐	MRI-based neuroimaging

Antibodies

Antibodies used	Flow cytometry anallyses were performed by using anti-CXCR4 (abcam; EPUMBR3, 1:25), anti-Lin-FITC (eBioscience; 17A2/RA3-6B2/M1-70/TER-119/RB6-8C5, 1:25), anti-c-kit-PE (eBioscience; 104D2, 1:25), anti-CD105-PE (eBioscience, MJ7/18, 1:25), anti-CD11b-FITC (eBioscience; M1/70, 1:25), anti-CD34-PE (Invitrogen; MEC14.7, 1:25), anti-CD45-PE (eBioscience; 30-F11, 1:25), or their corresponding isotype control antibodies (eBioscience).<br>Western blotting analyses were performed by using the antibodies for CXCR4 (abcam; UMB2, 1:1000), GAPDH (abcam; EPR16891, 1:5000), CD9 (abcam; EPR2949, 1:2000), CD63 (Santa Cruz; MX-49.129.5, 1:200), TSG101 (abcam; EPR7130(B), 1:1000), or actin (abcam; EPR16769, 1:5000).
Validation	All those antibodies used for Western blotting and flow cytometry in this report have been extensively used in the literature from previous studies, and validations are also shown in the product information sheets.

Animals and other organisms

Policy information about studies involving animals; ARRIVE guidelines recommended for reporting animal research

Laboratory animals	Male, 8 week-old C57BL/6 mice were used.
Wild animals	No wild animals were used.
Field-collected samples	No field-collected samples were used.
Ethics oversight	All animal experiments in this report were approved by the Animal Care and Use Committee of Huazhong University of Science and Technology and performed in compliance with the ''Guide for the Care and Use of Laboratory Animals'' (NIH publication) and all relevant ethical regulations for animal testing and research.

Note that full information on the approval of the study protocol must also be provided in the manuscript.

Human research participants

Policy information about studies involving human research participants

Population characteristics	Plasma samples were drawn from three patients (age range 49 - 74 yo; 2 males, one female) with underlying coronary artery disease and acute ST–elevation myocardial infarction during their initial hospital presentation, immediately prior to coronary intervention and stent placement. Control plasma samples were obtained from three patients (age range 64-78 yo; 1 male, 2 females) with underlying coronary artery disease (but without evidence of acute coronary syndrome) after elective percutaneous coronary intervention.
Recruitment	The patients were recruited at UAB medical center. Informed consent was obtained from all subjects.
Ethics oversight	Human studies were performed under UAB IRB protocols IRB-151201004 and IRB X130807012.

Note that full information on the approval of the study protocol must also be provided in the manuscript.

Flow Cytometry

Plots

Confirm that:

☒ The axis labels state the marker and fluorochrome used (e.g. CD4-FITC).

☒ The axis scales are clearly visible. Include numbers along axes only for bottom left plot of group (a 'group' is an analysis of identical markers).

☒ All plots are contour plots with outliers or pseudocolor plots.

☒ A numerical value for number of cells or percentage (with statistics) is provided.

Methodology

Sample preparation	The sample preparations, including their sources, are detailed in the report.
Instrument	Flow cytometry data were acquired on an LSRII (BD Biosciences, CA).
Software	The data were analyzed with FlowJo (Treestar, OR)
Cell population abundance	No cell sorting was performed. Sufficient cell numbers were used for flow cytometry analyses.
Gating strategy	The general gating strategy is described in the Methods, and we did not perform any analysis that needs sequential gating.

☐ Tick this box to confirm that a figure exemplifying the gating strategy is provided in the Supplementary Information.
